# Endophytic and rhizospheric bacteria associated with *Paspalum atratum* and its potential for plant growth promotion with different phosphate sources

**DOI:** 10.3389/fpls.2022.884716

**Published:** 2022-07-28

**Authors:** Ailton Ferreira de Paula, Felipe de Paula Nogueira Cruz, Naiana Barbosa Dinato, Paulo Henrique Marques de Andrade, Amanda Carolina Prado de Moraes, Waldomiro Barioni Junior, Alberto Carlos de Campos Bernardi, Bianca Baccili Zanotto Vigna, Alessandra Pereira Fávero, Paulo Teixeira Lacava

**Affiliations:** ^1^Laboratory of Microbiology and Biomolecules, Department of Morphology and Pathology, Biological and Health Sciences Center, Federal University of São Carlos, São Carlos, Brazil; ^2^Evolutionary Genetics and Molecular Biology Graduate Program, Biological and Health Sciences Center, Federal University of São Carlos, São Carlos, Brazil; ^3^Biotechnology Graduate Program, Exact and Technology Sciences Center, Federal University of São Carlos, São Carlos, Brazil; ^4^Embrapa Pecuária Sudeste, São Carlos, Brazil

**Keywords:** bacterial community, genetic resources, biological nitrogen fixation, indoleacetic acid, phosphate solubilization

## Abstract

The genus *Paspalum* belongs to the family Poaceae and has several species that are native to Brazil. The *Paspalum* Germplasm Bank (GB) of the Brazilian Agricultural Research Corporation comprises approximately 450 accessions from 50 species. Among these accessions, *Paspalum atratum* (BGP 308) has economic potential for forage purposes. However, the endophytic and rhizospheric microbial communities within this accession and their ability to promote plant growth remain unknown. The present study aimed to isolate the endophytic and rhizospheric bacteria associated with *P. atratum* and to assess their potential for plant growth improvement, so-called plant growth-promoting bacteria (PGPB). For the *in vitro* tests, the ability of nitrogen-fixing bacteria (NFB), phosphate solubilization (PS) and indoleacetic acid (IAA) production were evaluated. A total of 116 endophytic and rhizosphere bacteria were obtained from the isolation. In the *in vitro* tests, 43 (37.00%) of these isolates showed positive NFB, PS, and IAA results. These isolates were identified by 16S rDNA sequencing. The phosphate solubilization index (PSI) ranged from 2 to 3.61, all 43 strains performed biological nitrogen fixation and the IAA production ranged from 12.85 to 431.41 μg ml^−1^. Eight of these 43 isolates were evaluated *in vivo* in a greenhouse using *P. atratum* caryopsis. The pots were filled with soil prepared with three different phosphate sources and one control without phosphate. After growth, the plants were submitted to morphological, bromatological and chemical determination. Data were analyzed using analysis of variance (ANOVA) and principal component analysis (PCA). In the *in vivo* test, treatments 105 (*Pseudomonas* sp.) and 458 (*Pseudomonas* sp.) were the most significant for the crystalline phosphate source, 109 (*Bacillus* sp.) for the sedimentary phosphate source and, as for the soluble phosphate source most treatments that received bacterial isolates had higher phosphorus content in the dry matter than the uninoculated soluble phosphate control. The 105FCR (crystalline phosphate + *Pseudomonas* sp.), 109FSE (sedimentary phosphate + *Bacillus* sp.), and 110 FSE (sedimentary phosphate + *Enterobacter* sp.) treatments showed the best results for plant growth promotion. This work made it possible to determine the bacterial community associated with *P. atratum* (BGP308) and to obtain new potential plant growth-promoting strains.

## Introduction

Pasture is the main form of animal nutrition used for herds. Due to the potential for growth in different soils and climate conditions and requiring little management (Rodrigues et al., 2014), the genus *Urochloa* currently constitutes the majority of Brazilian pastures used for animal feed. The main problem in regard to *Urochloa* spp. is exposure to ecological imbalances owing to its low genetic variability (Compant et al., 2010). However, *Paspalum* is an important and highly diverse genus in the Poaceae family in the Americas (Novo et al., 2016), occurring throughout Brazil, Bolivia, Paraguay, Argentina, Chile and Uruguay (Zuloaga and Morrone 2005; Novo et al., 2019). Therefore, *Paspalum* spp. could potentially replace pastures composed of *Urochloa* spp. or could occupy areas where these grasses do not grow. Brazil is one of the largest meat producers and exporters globally; therefore, there is a need to research new sources and ways to carry out animal nutrition. According to the *Brazilian Association of Meat Exporting Industries* (ABIEC), Brazil exported approximately 8.50 million tons of beef in 2020 and 4.38 million tons in the first half of 2021, significantly contributing to Brazil’s economy (ABIEC, 2021).

Another critical point is that with the increase in cultivated areas, agrochemical use and environmental impacts also increase. Thus, alternative nonpolluting and more economical methods of promoting plant growth have gained greater attention (Coelho et al., 2019; Xiang et al., 2020).

Phosphate fertilizers are among the most commonly used agrochemicals in agriculture, and the lack of adequate levels of these fertilizers is a limiting factor for plant growth (Crous et al., 2019). Therefore, plants usually receive soluble phosphorus through industrialized fertilizers. In addition, some phosphate rocks can be applied for direct soil fertilization (Ahemad and Kibret, 2014). First, however, it is necessary to transform this phosphorus into a soluble form for plants. Phosphate-solubilizing bacteria (PSB) transform this phosphorus by chelation, ion exchange, and organic acid production (Khan et al., 2009; Afzal et al., 2019). Among the types of insoluble phosphate rocks are the sedimentary rocks of Arad (33.0% P_2_O_5_) and the crystalline rocks of Cajati/SP (5.0% P_2_O_5_) (Alves and Hagni, 2008; Ramos et al., 2009).

*Paspalum* accessions from the germplasm bank of Embrapa Pecuaria Sudeste have been evaluated for different uses, such as forage (Marcón et al., 2018) and turf (Souza et al., 2020). The studies developed with *Paspalum* plants from this GB have included characterization regarding shade stress (Barro et al., 2012), water stress (De Pezzopane et al., 2017), insect tolerance (Gusmão et al., 2016), and cryopreservation for new hybrid production (Dinato et al., 2018). However, studies related to the endophytic and rhizospheric bacterial microbiota diversity associated with this germplasm collection are still scarce.

Among the *Paspalum* accessions evaluated in the Brazilian Agricultural Research Corporation breeding program, BGP 308 from *P. atratum* Swallen is a promising accession for becoming a forage cultivar and for composing the preliminary studies of the endophytic and rhizospheric microbiota.

The analysis of the bacterial diversity associated with this species can indicate new microorganisms to be used for plant growth promotion in the forage *plant P. atratum*. Thus, to collaborate with works that seek alternative and sustainable ways to use phosphorus to avoid the environmental and economic impacts caused by the industrial process of obtaining industrial phosphorus.

The objective of this study was to search for phosphate-solubilizing bacteria, evaluate their ability to solubilize phosphate rocks, and promote plant growth.

The present study is the first report to examine phosphate rock in the nutrition of *P. atratum* plants, intermediated by cultivable endophytic and rhizospheric bacteria.

## Materials and methods

The work was divided into steps *in vitro* and *in vivo*. In the *in vitro* stage, there was isolation, identified and evaluated of the functional capacity of the plant growth-promoting bacterial strains. The three main characterizations performed were for phosphate solubilization (PS), nitrogen-fixing bacteria (NFB) and indoleacetic acid (IAA) production. In the *in vivo* stage, *P. atratum* plants were characterized in a morphological, nutritional and mineral manner (emphasizing phosphate solubilization), when inoculated or not with plant growth-promoting bacterial strains.

### Isolation of endophytic and rhizospheric bacteria

Samples (rhizospheric soils, roots, and leaves) from an adult plant of *P. atratum* BGP 308 (BRA 030078/VRcMmSv 14,525) were collected in August 2016 (dry season) and January 2017 (rainy season). Access BGP 308 belongs to the *Paspalum* germplasm bank, located at Brazilian Agricultural Research Corporation^1^ (21°57′42″S, 47°50′28″W, 860 m), São Carlos, SP, Brazil.

The endophytic bacterial community was isolated according to Araújo et al. (2014) and Bogas et al. (2015). Plant tissues were superficially disinfected by serial washes in 70% ethanol (EtOH) for 2 minutes, followed by 3% sodium hypochlorite for 3 minutes, 1 minute in 70% EtOH, and two rinses with sterile distilled water. Plant tissues were incubated in phosphate buffered saline (PBS) for 2 h at 28°C/200 rpm. Aliquots of 100 μl of decimal dilutions were inoculated in duplicate in plates containing tryptone soya agar (TSA) supplemented with Benlate (50 μg ml^−1^) to prevent fungal growth.

The isolation of rhizospheric bacteria was performed according to Mohite (2013) with modifications, in which the temperature used was 28°C, and aliquots of 100 μl of decimal dilutions were inoculated in duplicate in plates containing tryptic soy agar (TSA) supplemented with Benlate (50 μg ml^−1^). Bacterial cultures were preserved in tryptone soya broth (TSB) supplemented with glycerol (1:1) at −80°C until further study.

### Strain identification

Total DNA was extracted according to Aljanabi and Martinez (1997). The 16S gene was amplified using the primers V3F (5′-ACTCCTACGGGAGGCAGCAG-3′) and V6R (5′ ACAGCCATGCANCACCT 3′; Yang et al., 2016). Polymerase chain reaction (PCR) containing 60 ng of genomic DNA, 25 μl of Thermo Scientific PCR Master Mix (1.25 U of Taq polymerase enzyme, 1 × PCR buffer (200 mM Tris pH 8.4, 500 mM KCl), 50 mM MgCl_2_, and 1.25 mM dNTP) and 3 pmol of each primer was performed for the selected isolates. The reaction conditions consisted of an initial 95°C step for 3 min, followed by 31 cycles of 95°C for 30 s., 60°C for 30 s., 72°C for 1 min. and a final extension of 10 min at 72°C in a BioRad T100 thermocycler. The amplicons were examined by 0.7% agarose gel electrophoresis and purified by using a QIAquick (Qiagen) kit. The sequencing reactions were performed using the BigDye^®^ Terminator v3.1 Cycle Sequencing Kit (Thermo Fisher Scientific) and sequenced using an ABI 3730 DNA Analyzer. Sequences were analyzed by Sequencing Analysis 5.3.1 software using the Base Caller KB, and the low-quality sequences were visualized and edited using BioEdit software (Hall, 1999). The final sequence was subjected to BLASTn bacterial identification (Altschul et al., 1997; Maidak et al., 2000; Garrity et al., 2004).

The phylogenetic tree was obtained by multiple alignments of *P. atratum* endophytes and 16S rRNA sequences were retrieved from NCBI using the algorithm ClustalW and generated using MEGA X software (Kumar et al., 2018) using the maximum likelihood method in combination with a general time reversible model with 1,000 bootstrap replications as branch support (Saitou and Nei, 1987; Tamura et al., 2011). *Halobacterium* sp. (NR_113428.1) sequence was used as an outgroup.

### *In vitro* evaluation of plant growth-promoting bacteria

Bacteria isolated from *P. atratum* were qualitatively/quantitatively screened in triplicate for their ability to solubilize inorganic calcium phosphate. Strains were incubated in nutrient agar supplemented with Ca_3_(PO4)_2_ for 96 h at 28°C (Rodríguez and Fraga, 1999; Verma et al., 2001). As a result, the phosphate solubilization index (PSI) was calculated as the ratio of the total diameter (colony + halo zone) to the colony diameter and was classified as low (PSI < 2), medium (2 < PSI < 3), and high (PSI > 3; Silva Filho and Vidor, 2000; Pande et al., 2017).

The nitrogen-fixing bacteria (NFB) assay was carried out by growing the strains in a semisolid nitrogen-free medium twice for 72 h/28°C (Döbereiner et al., 1995). For the quantification of auxin production, the strains were grown in the broth tryptone de soy 10% medium supplemented with L-tryptophan for 72 h/28°*Ca.* This method was initially proposed by Bric et al. (1991) and adapted as a quantitative method (Husen, 2016).

### *In vivo* PGPB assay

Caryopsis of *P. atratum* were germinated and inoculated with eight different strains that presented PSI ≥ 2.0. The experiment was conducted in a greenhouse at the Brazilian Agricultural Research Corporation (see footnote 1), São Carlos, SP, Brazil. A randomized block design with three replications was used in a factorial scheme of 8 × 3 + 1 with pots containing 0.5 l and 4.5 l of soil.

### Soil preparation and treatments

The soil was prepared with three different sources of phosphate plus the control without phosphate, totaling 36 treatments and 108 pots. The three sources of P used were (1) soluble phosphate—triple granulated superphosphate (46.0% P_2_O_5_ soluble in neutral ammonium citrate + water), (2) sedimentary phosphate—Arad phosphate rock concentrate (33.0% P_2_O_5_ total), and (3) crystalline phosphate—Cajati phosphate rock concentrate (5.0% P_2_O_5_ total) with a dose of 200 mg kg^−1^ P or 458 mg kg^−1^ P_2_O_5_.

The chemical characteristics of the soil were determined according to Van Raij et al. (2001). Based on the soil analysis results, dolomitic limestone (total neutralizing power ratio, TNPR = 70%) was added to achieve a base saturation of 60% prior to transplanting. Then, the three phosphorus sources were applied at the transplanting of the seedlings, and the pots were fertilized with K_2_SO_4_ (60% K_2_O) until K reached 3% of the cation exchange capacity (CEC).

### Germination, seedling transplantation, and inoculations

The caryopses of the spikelets were removed and subjected to the disinfection process in a closed desiccator using the protocol described by Quesenberry et al. (2010). The caryopses were inserted into 16 × 100 mm test tubes containing Murashige and Skoog (MS) medium according to the Orbovic and Grosser (2006) seed germination protocol. After 14 days, the seedlings were removed from the test tube and inserted into a Falcon tube with 15 ml of bacterial suspension for 30 min at 28°C. The liquid MS medium was standardized at 10^9^ CFU/ml and used in this step. Thus, seedlings were transplanted into 500 ml pots containing limed soil without correction of nutrients. Five more inoculations were performed: in 500 ml pots, they received two more inoculations (15 and 30 days after transplant), and in 4.5 kg pots, they received three more inoculations (45, 60, and 75 days after transplant). Finally, the 6 ml volume of the standardized bacterial suspension was inoculated into the soil close to each plant’s root. The control was inoculated with phosphate buffered saline (PBS) solution without the bacterial isolate.

### Morphological, nutritional, and mineral analysis

The seedlings remained in the 500 ml pots for 43 days and were transplanted into the 4.5 kg pots for the four treatments. At 50 days after transplanting, the aerial parts of the plants were cut 15 cm from ground level and measured.

The aerial part samples were placed in an oven with forced circulation at 60°C for 72 h. After drying the samples and determining the dry leaf weight, the material was crushed in a Wiley mill with 1 mm sieves. The collected material was stored in a plastic bottle.

Thirty-two descriptors were evaluated: EP (phosphorus extract), EN (nitrogen extract), ECa (calcium extract), CP (crude protein), MM (mineral matter), LIG (lignin), Ca (calcium), Mg (magnesium), P (phosphorus), K (potassium), S (sulfur), Mn (manganese), Zn (zinc), N (nitrogen), SPAD (SPAD index), AFW (aerial fresh weight), ADW (aerial dry weight), LW (leaf width), NL (number of leaves), NT (number of tillers), ANT (presence of anthocyanin), LA (leaf area), EZn (zinc extract), DIV (*in vitro* digestibility), FDN (neutral fiber detergent), Ll (leaf length), PHe (plant height), Fe (iron), FDA (acid detergent fiber), MS (dry matter), EE (ether extract) and Cu (copper). Dry matter, crude protein, neutral detergent fiber, acid detergent fiber, lignin, and *in vitro* digestibility were determined using a near-infrared spectrometer (NIRS; Büchi Labortechnik, 2007) with a calibrated curve for *Paspalum*.

Based on Nogueira et al. (1998), the total nutrient content was determined. Nitrogen was determined in the extract of sulfuric digestion by the semimicro Khjeldhal method. The determination of K was made in the extract of nitro-perchloric digestion and determined by flame photometry. The other macronutrients (P, Ca, Mg, and S) and micronutrients (Cu, Fe, Mn, and Zn) were determined in the same nitro-perchloric extract and determined by induced plasma spectrometry (ICP–OES).

### Statistical analysis

Thirty-two traits (morphological, nutritional, and mineral) were analyzed. Data were analyzed using analysis of variance (ANOVA) and principal component analysis (PCA) with SAS^®^ 9.3 software (SAS Institute Inc, 2011).

## Results

### Isolation of endophytic and rhizospheric bacteria

In the rainy season isolation, the bacterial population ranged from 02 × 10^−1^ cfu gm^−1^ (leaf) to 43 × 10^−3^ cfu gm^−1^ (root), whereas in the dry season isolation, the bacterial population ranged from 05 × 10^−1^ cfu gm^−1^ (leaf) to 35 × 10^−2^ cfu gm^−1^ (rhizosphere; Table 1). In the rainy season, the soil had a pH of 5.4 (water) and 4.8 (CaCl_2_), and in the dry season, it was 5.6 (water) and 5.0 (CaCl_2_).

**Table 1:** Isolation of endophytic and rhizospheric bacteria from soil samples.

Samples	Isolation medium	Dilution	Amount of sample (ml)	Dilution factor (D)	Number of colony (24 h)	Mean cfu per 10 mg sample
Rhizosphere	TSA	10^−2^	0.1	10^2^	40	40 × 10^−2^
Root	TSA	10^−3^	0.1	10^3^	43	43 × 10^−3^
Leaf	TSA	10^−1^	0.1	10^1^	2	02 × 10^−1^

A total of 116 bacterial isolates were collected from *P. atratum* BGP 308, where 43 (37.00%) were obtained from rhizospheric soils, 42 (36.20%) from roots, and 31 (26.70%) from leaves. A total of 74 (63.70%) strains were isolated in the rainy season and 42 (36.20%) in the dry season.

### Identification and evaluation of the functional capacity of the plant growth-promoting bacterial strains

**Figure 1:**
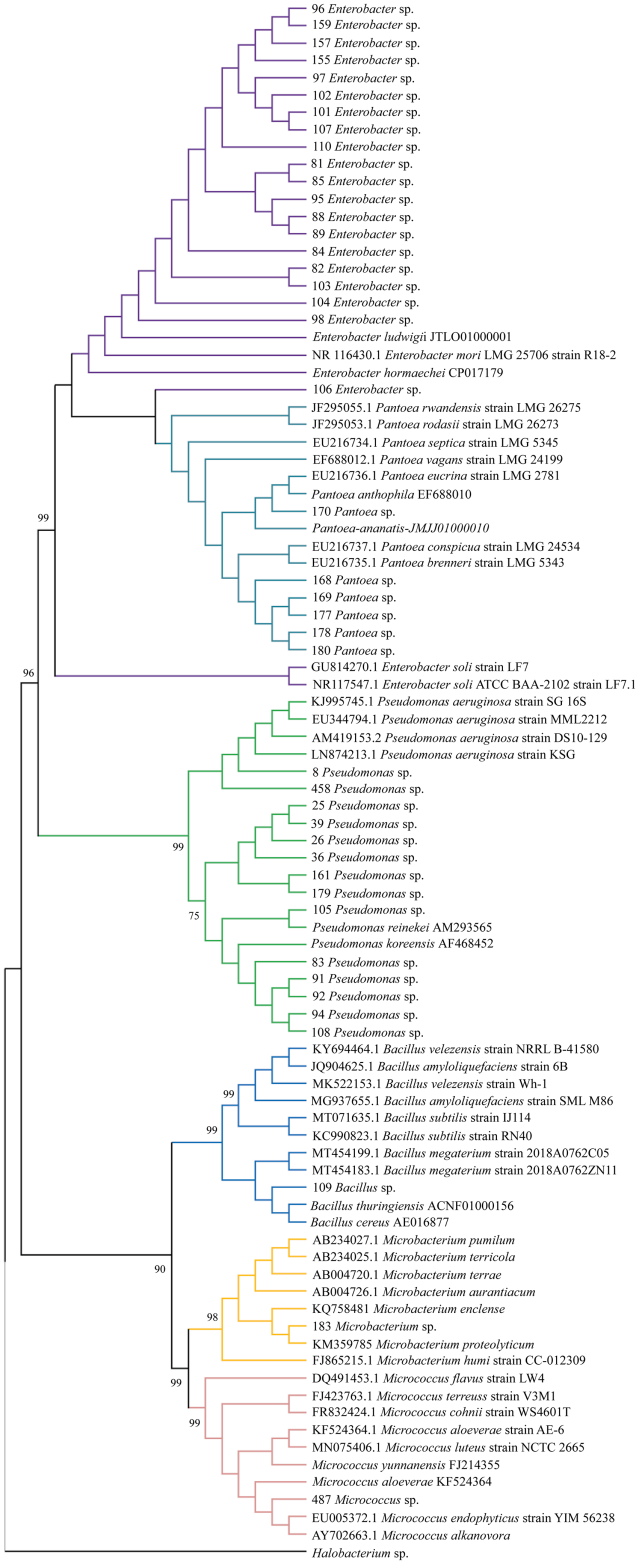
Consensus tree obtained from a maximum likelihood phylogenetic analysis using the general time reversible model (bootstrap with 1,000 replicates) based on 670 bp of the partial 16S rDNA gene.

Among the 116 strains obtained, 43 (37.00%) showed positive NFB, SF, and IAA results. From these, the strains that belonged to the genera *Enterobacter* (46.50%), *Pseudomonas* (32.50%), and *Pantoea* (13.90%) were the most abundant (Figure 1). On the other hand, *Bacillus*, *Microbacterium*, and *Micrococcus* strains represented only 6.90%. The phylogenetic analysis showed highly significant support (>98%) for the groups formed of samples from each genus and its correspondent reference sequences for the genus. Moreover, nodes forming a group with all the samples from genera *Enterobacter*, *Pantoea* and *Pseudomonas* and another group with all the samples from genera *Bacillus*, *Microbacterium* and *Micrococcus* also showed significant support (>90%), showing the relation among genera identified in this study (Figure 1).

The phosphate solubilization index (PSI) ranged from 2 to 3.61 (Table 2). Strains 103, 89, and 102, which are root endophytes and belong to the *Enterobacter* genus, showed the best results, with PSI values of 3.61, 3.58, and 3.56, respectively. All 43 strains in Table 2 performed biological nitrogen fixation, characterized by a semisolid nitrogen-free medium. The IAA production ranged from 12.85 to 431.41 μg ml^−1^. The endophytic leaf strain 170 (*Pantoea* sp.) showed the highest IAA production (431.41 μg ml^−1^), followed by 18 *Enterobacter* spp.; endophytes from roots presented 105.05–263.74 μg ml^−1^ of IAA production.

**Table 2:** Molecular identification of the genera of the 43 bacterial strains that showed positive results for the phosphate solubilization index (PSI), nitrogen-fixing bacteria (NFB), and indole acetic acid (IAA) production.

Sample	Bacterial genus	Strains code	Accession number (GenBank)	PSI	NFB	IAA (μg.ml^–1^)
Root	*Enterobacter* spp.	103	MK521286	3.61	+	165.29
Root	*Enterobacter* spp.	89	MK521276	3.58	+	222.62
Root	*Enterobacter* spp.	102	MK521285	3.56	+	134.54
Leaf	*Pantoea* spp.	169	MK521301	3.41	+	66.04
Leaf	*Micrococcus* spp.	487	MK521314	3.38	+	47.38
Leaf	*Pantoea* spp.	180	MK521306	3.36	+	61.09
Root	*Enterobacter* spp.	84	MK521273	3.28	+	164.52
Leaf	*Pantoea* spp.	178	MK521304	3.18	+	52.33
Root	*Enterobacter* spp.	101	MK521284	3.10	+	133.37
Root	*Enterobacter* spp.	106	MK521289	3.10	+	105.05
Leaf	*Pantoea* spp.	177	MK521303	3.10	+	59.32
Leaf	*Pantoea* spp.	170	MK521302	3.06	+	431.41
Root	*Enterobacter* spp.	85	MK521274	2.99	+	204.32
Leaf	*Pantoea* spp.	168	MK521300	2.97	+	28.04
Root	*Enterobacter* spp.	88	MK521275	2.88	+	148.75
Root	*Enterobacter* spp.	155	MK521296	2.88	+	263.74
Root	*Enterobacter* spp.	107	MK521290	2.87	+	131.33
Root	*Enterobacter* spp.	110	MK521293	2.85	+	87.6
Root	*Enterobacter* spp.	104	MK521287	2.81	+	166.95
Root	*Enterobacter* spp.	82	MK521271	2.77	+	187.87
Root	*Enterobacter* spp.	81	MK521270	2.76	+	216.78
Root	*Enterobacter* spp.	98	MK521283	2.75	+	187.73
Root	*Enterobacter* spp.	97	MK521282	2.7	+	135.85
Root	*Pseudomonas* spp.	83	MK521272	2.69	+	23.25
Root	*Pseudomonas* spp.	94	MK521279	2.65	+	25.24
Rhizosphere	*Pseudomonas* spp.	458	MK521308	2.62	+	65.12
Root	*Pseudomonas* spp.	91	MK521277	2.62	+	21.64
Root	*Enterobacter* spp.	157	MK521297	2.62	+	142.23
Root	*Pseudomonas* spp.	92	MK521278	2.61	+	21.71
Root	*Bacillus* spp.	109	MK521292	2.58	+	18.26
Leaf	*Pseudomonas* spp.	161	MK521299	2.55	+	72.15
Root	*Pseudomonas* spp.	108	MK521291	2.47	+	14.54
Root	*Enterobacter* spp.	95	MK521280	2.46	+	205.29
Root	*Enterobacter* spp.	159	MK521298	2.45	+	158.29
Root	*Enterobacter* spp.	96	MK521281	2.44	+	88.12
Leaf	*Pseudomonas* spp.	179	MK521305	2.39	+	12.85
Leaf	*Microbacterium* spp.	183	MK521307	2.23	+	20.79
Rhizosphere	*Pseudomonas* spp.	8	MK521261	2.20	+	16.09
Root	*Pseudomonas* spp.	105	MK521288	2.18	+	35.85
Rhizosphere	*Pseudomonas* spp.	26	MK521263	2.02	+	27.75
Rhizosphere	*Pseudomonas* spp.	25	MK521262	2.00	+	44.85
Rhizosphere	*Pseudomonas* spp.	36	MK521264	2.00	+	24.39
Rhizosphere	*Pseudomonas* spp.	39	MK521265	2.00	+	27.33

All strains belonging to *Microbacterium*, *Micrococcus*, *Pantoea*, *Bacillus*, and *Enterobacter* were endophytes, while the *Pseudomonas* strains were either endophytic or rhizospheric. Among the 43 strains selected for the *in vitro* tests, six were isolated from the rhizosphere, 27 from the root, and 10 from the leaf (Table 3).

**Table 3:** Bacterial genus, number of strains, origin and isolation period of strains of BGP 308 from *P. atratum* that were positive for phosphate solubilization, biological nitrogen fixation, and indole acetic acid production.

Origin and isolation period of strains					
Bacterial genus					
	Rhizosphere	Rhizosphere	Root	Root	Leaf	Leaf
	(Dry)	(Rainy)	(Dry)	(Rainy)	(Dry)	(Rainy)
*Bacillus* spp.	–	–	1	–	–	–
*Enterobacter* spp.	–	–	20	–	–	–
*Microbacterium* spp.	–	–	–	–	1	–
*Micrococcus* spp.	–	–	–	–	–	1
*Pantoea* spp.	–	–	–	–	6	–
*Pseudomonas* spp.	5	1	6	-	2	-

Traces means no microorganism was selected for a given condition.

### Plant growth-promotion assay

In the plant growth promotion assay, the selected strains belonged to the rhizosphere, root and leaf. The PSI ranged from 2 to 3.61, and all were positive for NFB and IAA. The rhizospheric bacteria selected for the *in vivo* test were Isolates 25 (*Pseudomonas* sp.) and 458 (*Pseudomonas* sp.), and the endophytic bacteria were Isolates 103 (*Enterobacter* sp.), 105 (*Pseudomonas* sp.), 109 (*Bacillus* sp.), 110 (*Enterobacter* sp.), 161 (*Pseudomonas* sp.), and 170 (*Pantoea* sp.).

The triple interaction [Source of Phosphorus (3) vs. Isolate (9) vs. Cut (3)] for Phosphorus extract (P) was significant with *p*-value = 0.0023.

**Figure 2:**
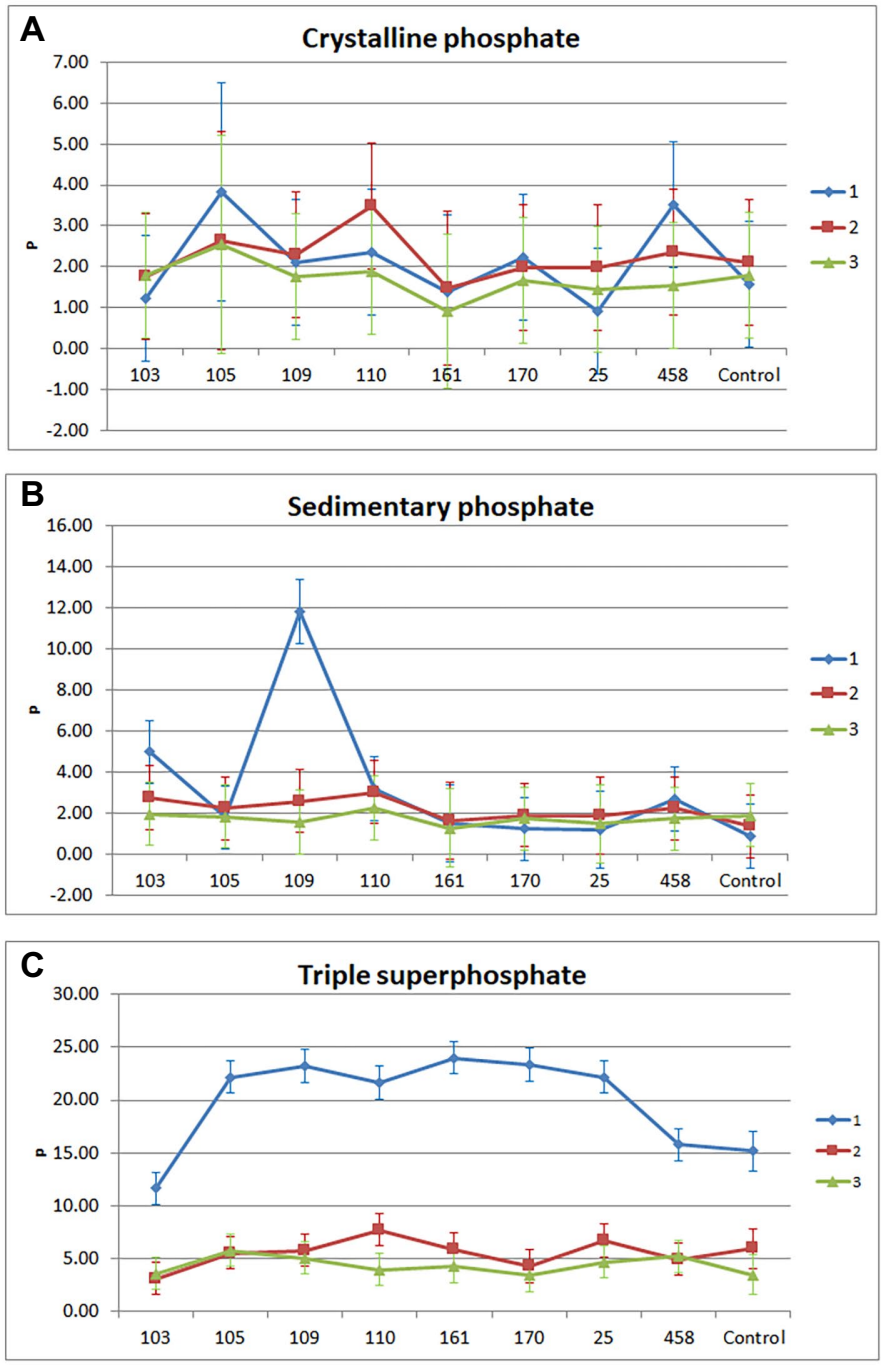
Triple interaction (source of phosphorus vs. isolate vs. cut). Treatments that received crystalline **(A)**, sedimentary **(B)**, and soluble **(C)** phosphate as a source of phosphorus.

The P extract showed that in treatments with crystalline phosphate, the mean phosphorus content in dry matter ranged from 0.90 to 3.82 kg ha^–1^, considering treatments and cuts (Figure 2). The treatments that received strains 105 (*Pseudomonas* sp.) and 458 (*Pseudomonas* sp.) had a higher phosphorus content than the control crystalline phosphate in the first cut and showed a drop in the second third cut. The treatment containing Strain 110 (*Enterobacter* sp.) presented a high level of phosphorus in the second cut compared to the control. Within this treatment, P extract increased in the second cut, and decreased it in the first and third cut. Despite the numerical variations, in the phosphorus content, there was no statistical difference (*p* > 0.05). In treatments with sedimentary phosphate, the mean phosphorus content in dry matter ranged from 0.89 to 11.82 kg ha^–1^, considering treatments and cuts. The treatment that received the bacterial isolate 109 (*Bacillus* sp.) had a higher phosphorus content than the sedimentary phosphate control in the first cut (*p* > 0.05). Within this treatment, the phosphorus content was higher in the first cut, followed by the second and third cut (*p* ≤ 0.05). The treatment that received strain 103 (*Enterobacter* sp.) also showed an increase in phosphorus content in the first cut. In the other treatments there were no (*p* > 0.05) significant changes in the phosphorus content in the cuts.

In treatments with soluble phosphate, the mean phosphorus content in dry matter ranged from 3.14 to 24.00 kg ha^–1^, considering treatments and cuts. Regardless of treatment, the phosphorus content was higher in the first cut (*p* ≤ 0.05). In this cut, the lowest phosphorus content in the dry matter was in the treatment that received the strain 103 (*Enterobacter* sp.) and the highest was in the treatment that received the strain 161 (*Pseudomonas* sp.) with 11.65 and 24.00 kg ha^–1^, respectively. The treatment that received the bacterial isolate 110 (*Enterobacter* sp.) showed a significant difference (*p* ≤ 0.05) between the three cuts, for the phosphorus content, with 21.66, 7.73 and 3.98 kg ha^–1^, respectively. The phosphorus content, in cut1, for the control of uninoculated soluble phosphate was 15.21 kg ha^–1^. In cuts 2 and 3, the maximum phosphorus content reached was 7.73 kg ha^–1^, regardless of the treatments.

The results of the principal component analysis (PCA) showed that Component 1 (PRIN1 = 65.31%) and Component 2 (PRIN2 = 8.52%) explained 73.83% of the variance observed. A total of 22 variables were significant (correlation > 50%) in the discrimination of treatments (Table 4) among the 32 variables used.

**Table 4:** Contribution of morphological descriptors for principal component analysis.

Variable	Variable	PCR1	PCR2
Aerial fresh weight	AFW	0.965	0.082
Aerial dry weight	ADW	0.962	0.059
Calcium extract	ECa	0.954	0.029
Nitrogen extract	EN	0.954	0.117
Leaf area	LA	0.942	0.085
Number of tillers	NT	0.928	−0.111
Magnesium	Mg	0.904	0.254
Number of leaves	NL	0.892	−0.095
Phosphorus extract	EP	0.889	−0.088
Sulphur	S	0.885	0.235
Phosphorus	P	0.857	0.177
Calcium	Ca	0.792	0.223
Mineral matter	MM	0.61	0.488
Leaf width	LW	0.578	0.185
Presence of anthocyanin	ANT	0.557	−0.387
Manganese	Mn	0.512	−0.021
Zinc extract	EZn	0.405	0.172
*In vitro* digestibility	DIV	0.279	−0.424
Neutral fiber detergent	FDN	0.179	−0.804
Leaf length	Ll	0.029	0.505
Plant height	PHe	−0.053	0.399
Iron	Fe	−0.147	0.364
Acid detergent fiber	FDA	−0.23	−0.613
Dry matter	MS	−0.232	−0.664
Ether extract	EE	−0.373	0.169
Copper	Cu	−0.433	0.018
Lignin	LIG	−0.587	0.51
Zinc	Zn	−0.658	−0.135
Crude protein	CP	−0.67	0.53
Nitrogen	N	−0.67	0.532
SPAD index	SPAD	−0.805	0.17
Potassium	K	−0.877	−0.058

**Figure 3:**
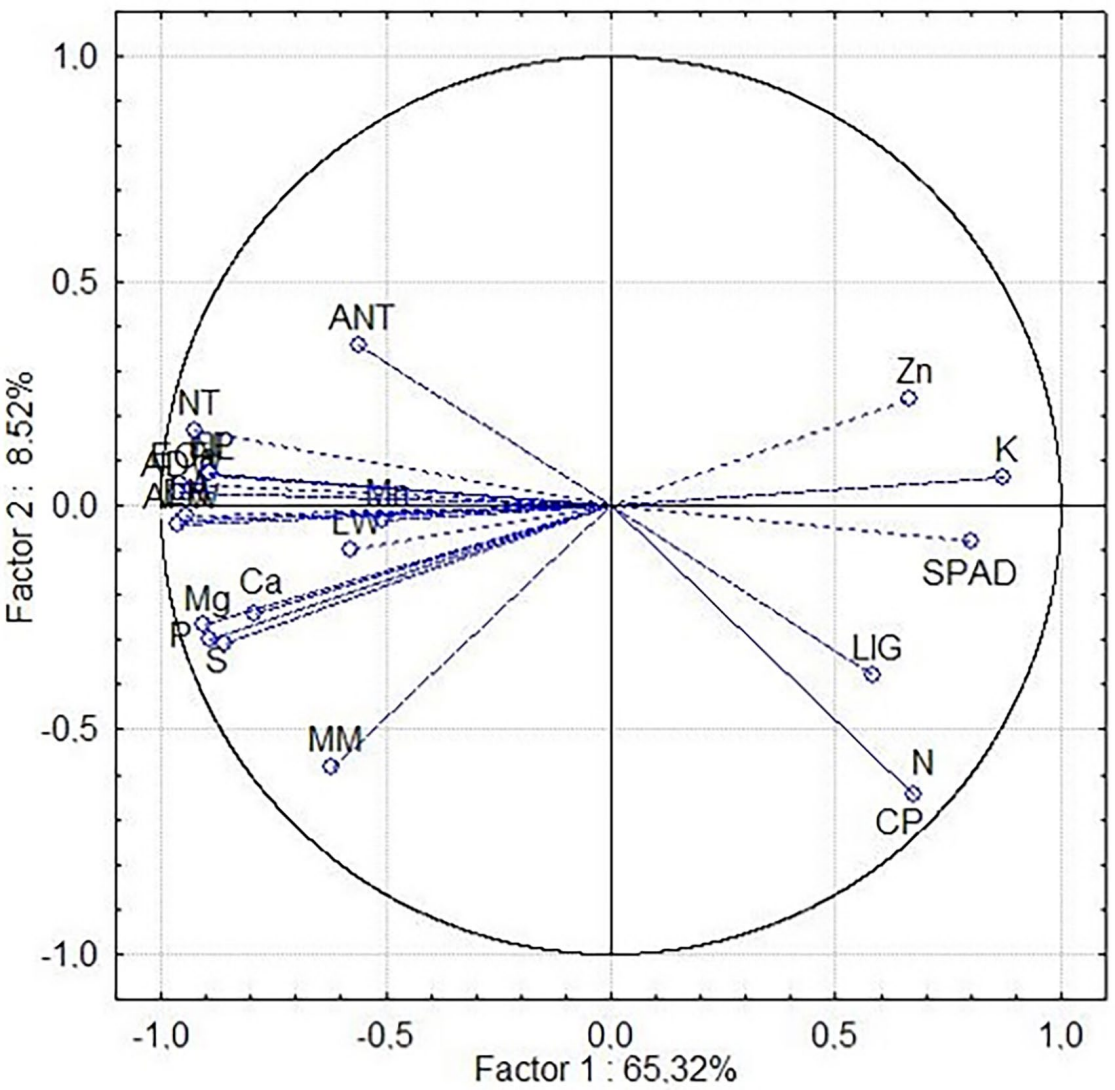
Principal component analysis of the 22 descriptors evaluated. Correlation between the evaluated descriptors. Phosphorus extract (EP), nitrogen extract (EN), calcium extract (ECa), crude protein (CP), mineral matter (MM), lignin (LIG), calcium (Ca), magnesium (Mg), phosphorus (P), potassium (K), sulfur (S), manganese (Mn), zinc (Zn), nitrogen (N), SPAD index (SPAD), aerial fresh weight (AFW), aerial dry weight (ADW), leaf width (LW), number of leaves (NL), number of tillers (NT), presence of anthocyanin (ANT), and leaf area (LA).

When evaluating the phosphate source used in the experiment, the analyzed variables divided the treatments into two groups (Figure 3), directed by Principal Components 1 (PRIN1) and 2 (PRIN2). The treatments that received soluble phosphate are distributed on the left side of the graph. In contrast, the treatments without phosphate (control) and those receiving sedimentary and crystalline phosphate are distributed on the right side. For the most significant morphological, mineral and bromatological variables, the principal component analysis showed that Principal Component 1 had significant associations with the SPAD index (SPAD), potassium (K), zinc (Zn), crude protein (CP), lignin (LIG) and nitrogen (N) (Figure 2). Furthermore, these variables were responsible for grouping the nine treatments (103 FSO, 105 FSO, 109 FSO, 110 FSO, 161 FSO, 170 FSO, 25 FSO, 458 FSO, and CAFSO) that received the soluble phosphate source in Group 1.

Principal Component 2 showed strong associations with aerial fresh weight (AFW), aerial dry weight (ADW), leaf width (LW), number of leaves (NL), number of tillers (NT), anthocyanin (ANT), area leaf (LA), phosphorus extract (EP), calcium extract (ECa), calcium (Ca), magnesium (Mg), phosphorus (P), sulfur (S), manganese (Mn), nitrogen (EN) and mineral matter (MM) (Figure 2). These variables grouped the 27 treatments (103FCR, 103FSE, 103SF, 105FCR, 105FSE, 105SF, 109FCR, 109FSE, 109SF, 110FCR, 110FSE, 110SF, 161FCR, 161FSE, 161SF, 170FCR, 170FSE, 170SF, 25FCR, 25FSE, 25SF, 458FCR, 458FSE, 458SF, CAFCR, CAFSE and CASF) that did not receive the soluble phosphate source in Group 2.

Tables 5, 6 describe the order of descriptors that most contributed to the morphological variation observed in Principal Component 1 (PRIN 1) of the principal component analysis (PCA) of all treatments evaluated in this work. Analyzing the most important descriptors (SPAD, K, Zn, CP, LIG and N), it can be observed that the treatments that received soluble phosphate presented lower values (Tables 5, 6) for these descriptors, and this located these treatments in group 1 (Figure 3). The inverse of values for these descriptors are represented in the procedures that they located in group 2.

**Table 5:** Values of extracts and morphological descriptors evaluated on average.

TRAT	EP	EN	ECa	AFW	ADW	LW	NL	NT	ANT	LA	SPAD
103FCR	4.77	143.39	53.00	45.58	7.77	47.47	76.00	21.00	0.00	1728.29	94.66
103FSE	18.41	117.97	65.46	38.18	6.34	45.15	64.33	16.67	0.67	1572.39	98.48
103FSO	9.73	229.95	101.51	80.61	12.24	47.61	102.67	29.00	0.00	2819.05	78.35
103SF	7.77	197.23	92.91	57.63	10.02	47.37	90.33	24.00	0.00	2075.62	92.30
105FCR	9.00	242.43	139.71	67.77	13.23	48.40	75.00	26.00	0.00	2520.89	93.10
105FSE	5.90	128.34	55.66	38.06	6.70	42.62	57.33	18.00	0.00	1460.29	91.15
105FSO	33.49	267.02	170.67	86.24	15.65	46.88	116.00	39.67	2.00	3250.76	79.17
105SF	6.94	162.80	78.76	49.25	8.80	44.34	80.67	24.67	0.67	1877.44	89.00
109FCR	6.18	168.98	73.43	51.32	9.38	43.97	92.67	26.00	0.00	2025.18	92.75
109FSE	15.99	187.61	100.17	55.86	10.12	47.98	81.33	21.33	0.00	2401.46	94.24
109FSO	34.11	257.14	144.78	85.97	14.12	48.31	105.00	31.33	1.33	2975.36	77.53
109SF	3.18	136.65	40.71	41.58	7.12	47.39	73.33	18.67	0.33	1265.03	96.55
110FCR	7.73	152.25	83.83	48.22	8.49	43.36	81.00	23.67	0.00	1657.16	93.17
110FSE	8.51	177.87	88.64	65.35	9.81	48.32	97.67	27.33	0.67	2482.98	82.77
110FSO	33.38	237.84	150.67	71.60	12.61	48.42	113.00	28.33	2.33	2513.11	80.52
110SF	6.90	170.86	78.08	52.62	8.98	43.92	75.67	23.00	1.33	1937.38	91.54
161FCR	3.79	95.33	44.01	34.25	5.31	43.24	57.50	18.00	0.50	1247.10	85.35
161FSE	4.47	132.72	46.03	43.94	7.05	43.61	77.00	21.00	0.50	1702.60	95.25
161FSO	34.12	258.69	156.58	82.58	13.55	47.19	111.00	32.67	0.00	2878.92	81.07
161SF	7.30	178.25	84.54	55.00	9.25	47.27	78.50	22.00	0.50	2141.04	98.53
170FCR	5.90	154.12	69.07	48.89	8.32	46.08	90.33	25.67	0.00	1721.76	88.71
170FSE	4.92	123.40	48.91	46.76	6.68	42.53	63.33	17.33	0.00	1590.86	93.22
170FSO	31.04	271.82	149.72	82.53	14.90	49.22	131.00	38.00	1.67	2813.21	82.25
170SF	5.32	126.78	63.86	38.22	6.88	45.64	80.67	22.00	0.67	1379.32	92.59
25FCR	4.33	109.01	39.24	39.29	5.90	40.08	73.67	20.00	0.33	1324.59	89.14
25FSE	4.59	133.15	60.82	39.23	6.67	44.39	73.00	21.00	0.00	1328.45	94.39
25FSO	33.61	259.85	167.92	78.28	14.31	45.54	99.33	30.67	1.33	2961.34	77.20
25SF	6.38	175.93	81.28	53.41	9.49	48.62	75.00	20.67	0.00	2114.69	98.42
458FCR	7.44	183.05	91.76	52.41	9.68	45.13	77.33	22.67	0.67	1786.17	94.45
458FSE	6.68	163.90	78.93	52.02	8.87	43.85	79.67	23.67	1.33	2039.63	84.69
458FSO	25.92	229.01	117.09	74.05	12.72	48.32	109.33	29.33	1.00	2522.91	85.12
458SF	9.12	192.69	92.46	58.94	10.59	48.10	80.33	26.00	0.33	2017.38	95.50
CAFCR	5.46	138.77	56.43	43.30	7.32	46.94	73.00	20.33	0.00	1598.96	98.06
CAFSE	4.18	104.83	38.57	35.93	5.70	42.27	76.67	16.00	0.00	1228.91	94.01
CAFSO	24.67	285.21	167.37	76.06	14.86	48.34	104.00	35.50	0.50	2681.90	83.67
CASF	5.48	146.56	53.94	50.84	8.16	48.84	82.67	22.00	1.00	1722.92	90.53

Phosphorus extract (EP), nitrogen extract (EN), calcium extract (ECa), aerial fresh weight (AFW), aerial dry weight (ADW), leaf width (LW), number of leaves (NL), number of tillers (NT), presence of anthocyanin (ANT), leaf area (LA) and SPAD index (SPAD).

**Table 6:** Values of mineral and bromatological descriptors evaluated on average.

TRAT	MM	Ca	Mg	P	S	Mn	K	Zn	LIG	CP	N
103FCR	9.16	9.26	10.22	0.80	4.96	63.98	11.15	14.80	3.77	12.46	19.93
103FSE	8.33	7.04	7.96	0.63	4.11	71.27	14.18	13.32	3.39	11.68	18.69
103FSO	9.63	10.67	14.17	3.17	6.79	95.04	8.49	8.55	2.95	11.97	19.16
103SF	8.87	8.07	9.66	0.78	4.42	58.83	11.20	14.58	2.88	11.44	18.31
105FCR	8.77	10.21	11.53	0.68	5.03	67.23	10.15	11.81	6.18	11.14	17.83
105FSE	8.63	8.33	9.63	0.88	4.69	96.74	14.56	17.56	4.40	11.99	19.19
105FSO	9.02	10.50	12.71	1.95	5.86	76.82	7.14	9.41	2.04	10.12	16.19
105SF	8.49	8.73	9.40	0.78	4.67	78.90	10.99	12.54	2.80	11.48	18.36
109FCR	8.04	7.81	9.27	0.66	4.08	61.19	10.45	13.92	2.80	11.25	18.00
109FSE	8.57	9.56	12.17	1.43	6.47	73.56	8.93	11.59	2.89	11.44	18.31
109FSO	9.53	9.97	11.79	2.16	5.79	83.02	7.35	8.33	1.97	10.95	17.52
109SF	8.36	5.61	6.96	0.45	3.28	62.85	9.15	8.67	3.75	12.07	19.32
110FCR	8.69	9.82	10.06	0.92	4.73	69.86	10.80	14.49	3.51	11.14	17.83
110FSE	8.80	8.99	10.28	0.86	4.54	69.27	9.35	15.71	3.30	11.29	18.07
110FSO	9.17	11.70	14.84	2.37	6.30	88.98	8.70	8.05	2.35	11.57	18.51
110SF	8.25	8.76	9.75	0.77	4.26	80.24	10.38	14.44	3.11	11.94	19.10
161FCR	8.27	8.22	7.90	0.71	4.09	109.41	12.81	12.77	4.15	11.15	17.84
161FSE	8.85	6.78	8.47	0.65	4.41	62.53	11.84	13.30	4.55	11.98	19.17
161FSO	9.37	11.02	13.54	2.18	6.40	120.32	7.29	9.46	2.38	11.43	18.29
161SF	9.13	9.09	9.91	0.79	4.77	49.85	9.61	12.88	5.90	12.04	19.26
170FCR	8.23	8.25	9.28	0.71	4.33	76.29	11.45	17.27	3.37	11.53	18.45
170FSE	8.60	7.57	8.84	0.76	4.17	71.28	13.78	14.96	4.96	11.85	18.95
170FSO	9.09	8.88	11.51	1.71	6.12	76.42	7.03	7.78	2.13	10.81	17.30
170SF	8.85	9.36	9.33	0.78	4.44	63.97	11.76	14.64	2.90	11.56	18.50
25FCR	8.54	7.08	8.03	0.79	3.90	70.75	13.53	13.18	3.52	12.19	19.50
25FSE	8.95	8.62	9.28	0.64	4.35	58.06	9.41	11.27	4.21	11.64	18.63
25FSO	8.75	11.08	13.82	2.09	6.66	89.59	7.16	9.45	1.80	10.86	17.37
25SF	8.28	8.62	10.21	0.68	4.51	70.72	11.38	17.95	3.30	11.71	18.74
458FCR	9.08	9.12	9.83	0.75	4.88	75.66	9.69	12.98	3.86	11.59	18.55
458FSE	8.65	8.82	10.22	0.77	4.64	84.32	10.50	18.55	3.06	11.38	18.20
458FSO	9.42	8.82	12.22	1.95	5.74	71.30	7.71	10.68	2.57	11.28	18.05
458SF	8.09	8.59	10.79	0.86	4.55	85.10	10.46	16.38	2.02	11.28	18.05
CAFCR	8.49	7.89	8.86	0.76	4.15	43.95	12.02	14.16	3.62	11.98	19.17
CAFSE	8.69	7.28	8.03	0.78	3.73	51.14	11.87	14.85	3.22	11.81	18.89
CAFSO	9.03	10.17	12.82	1.58	6.26	118.00	7.28	11.60	2.33	11.24	17.98
CASF	8.21	6.77	8.40	0.70	4.00	64.40	12.24	16.71	2.54	11.54	18.46

Mineral matter (MM), calcium (Ca), magnesium (Mg), phosphorus (P), sulfur (S), manganese (Mn), potassium (K), zinc (Zn), lignin (LIG), crude protein (CP) and nitrogen (N).

**Figure 4:**
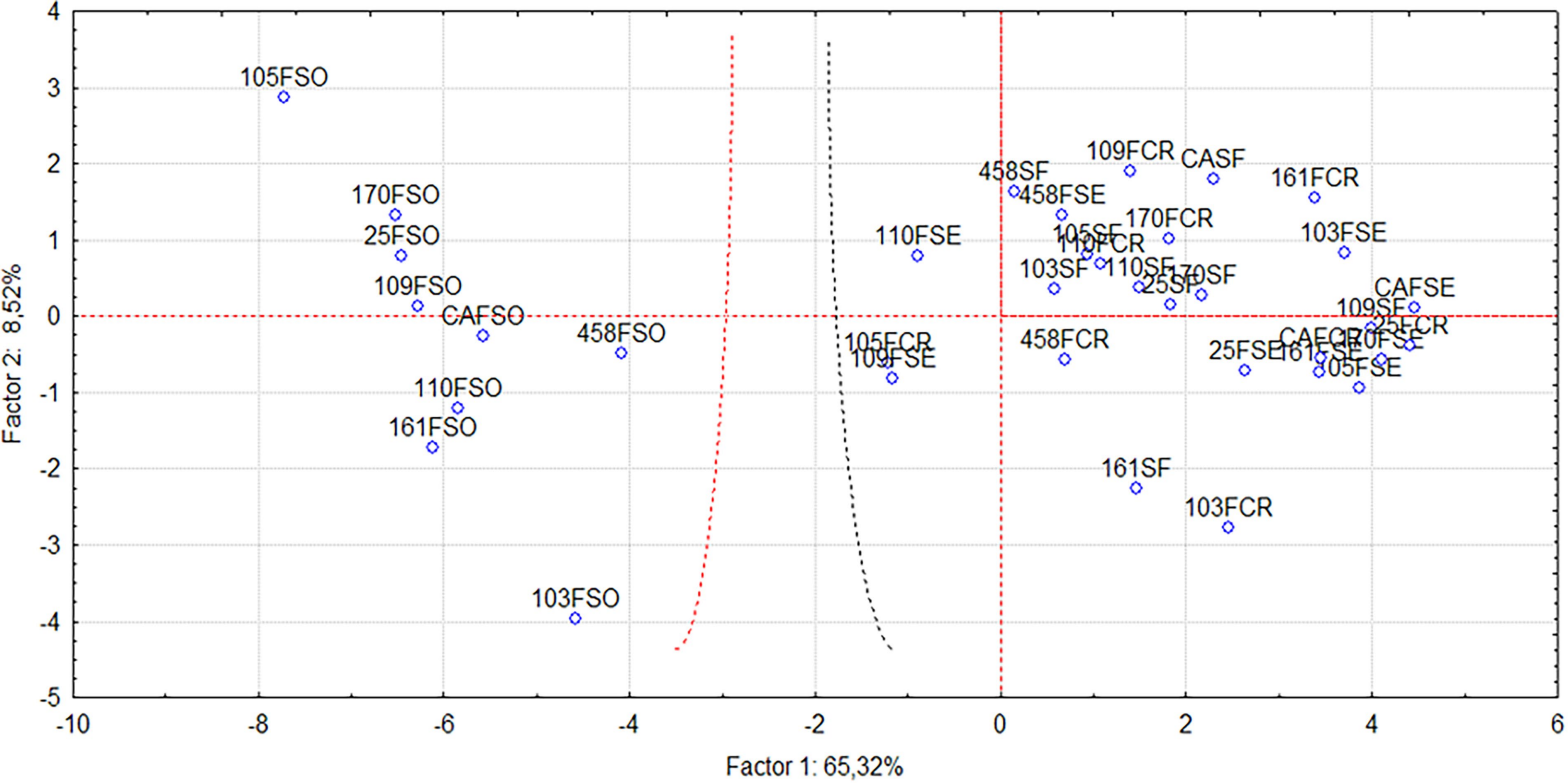
Biplot graph resulting from the 36 treatments obtained by principal component analysis considering the 22 descriptors for Principal Components 1 and 2. Control (CA), crystalline phosphate (FCR), sedimentary phosphate (FSE), phosphate-free (SFO), and soluble phosphate (FSO). The number that precedes the treatment refers to the strain code.

In Figure 4, the principal component graph shows the two groups and treatments closest to those receiving phosphorus from the soluble phosphate source. The group on the right side of the graph belongs to treatments with soluble phosphate. In contrast, the group to the left and central part of the graph belongs to treatments containing sedimentary phosphate, crystalline phosphate, and no phosphate (control). Any treatment that received sedimentary and crystalline phosphate was not observed, composing the group of those that received the soluble phosphate. Nevertheless, the ones that came closest were the 105FCR (crystalline phosphate + *Pseudomonas* sp.), 109FSE (sedimentary phosphate + *Bacillus* sp.), and 110FSE (sedimentary phosphate + *Enterobacter* sp.) treatments.

## Discussion

In the production of pastures for animal nutrition, there is great concern regarding the cultivation of plants. For cultivation, plants obtain most of their nutrients from some industrialized source. In 2018, Brazil consumed 852.4 thousand tons of triple superphosphate (largest consumer on a global scale) and 2,437.7 thousand tons of ammonium phosphate (third consumer on a global scale). For nitrogen, phosphorus, and potassium (NPK) fertilizer, Brazil consumed 15507.1 thousand tons and imported 13338.7 thousand tons, the largest importer of NPK on a worldwide scale (International Fertilizer Industry Association, 2021). Microorganisms can provide nutrients from alternative and nonindustrialized sources to plants. The main focus of this work was to address phosphate solubilization by plant growth-promoting bacteria (PGPB).

Studies involving the endophytic and rhizospheric microbiota of *Paspalum* accessions are scarce. Zhao et al. (2021) isolated endogenous *Enterobacter* strains from *Paspalum vaginatum* and evaluated their influence on promoting salt tolerance in the plant. Pérez and Martínez (2016) isolated and identified endophytic bacteria that is resistant to mercury associated with *P. arundinaceum* in Mina Santa Cruz, Bolivar., Colombia, aiming to obtain plant growth-promoting strains with potential for phytoremediation of mercury-contaminated soil. Amaral et al. (2021) isolated and characterized plant growth-promoting rhizobacteria (PGPR) from 10 genotypes of *Paspalum* and evaluated the effect of inoculation on *P. regnellii*, *P. atratum*, and *P. malacophyllum*. The genotypes were also collected from the *Paspalum* germplasm bank at the Brazilian Agricultural Research Corporation; however, the genotype of *P. atratum* studied was cv. *Pojuca* BGP 098. For studies involving phosphate solubilization in *Paspalum* with potential for forage, this work is the first to examine phosphate rock in the nutrition of *P. atratum* plants, intermediated by cultivable endophytic and rhizospheric microorganisms.

Based on the present study results, the bacterial population was higher in the rhizosphere and the root. The rhizosphere exhibits different physical and chemical characteristics than nonrhizospheric soil, as plants can produce root exudates, which provide bacterial nutrition and make this environment favorable for bacterial colonization (Wu et al., 2018). In the case of endophytic bacteria, the root is the main entrance way for microorganisms in plants (White et al., 2014). Thus, it is also expected that the roots present a greater population of microorganisms than other plant segments, such as stems and leaves. Abedinzadeh et al. (2019) found similar data, in which the population size was 3.4 ± 2.12 × 10^6^ and 6.8 ± 1.20 × 10^3^ for rhizospheric and endophytic bacteria in maize plants.

Among the 116 strains isolated, 43 showed positive results for NFB, SF, and IAA. The 43 strains belonged to Proteobacteria, Firmicutes, and Actinobacteria. Genetic diversity studies have reported that these phyla are both endophytic and rhizospheric (Rosenblueth and Martínez-Romero, 2006; Pisa et al., 2011; Prabha et al., 2018) and generally present strains with potential for plant growth promotion.

In this work, through *in vitro* tests, the main strains that showed the potential to promote plant growth belonged to *Bacillus*, *Enterobacter*, *Microbacterium, Micrococcus*, *Pantoea*, and *Pseudomonas* (Amaral et al., 2021). The main PGPB isolated from *Paspalum* genotypes were *Acinetobacter*, *Bacillus*, *Cupriavidus*, *Dyadobacter*, *Enterobacter*, *Paraburkholderia*, *Pseudomonas*, and *Rhizobium*.

In the *in vivo* test, treatments were separated into two groups. Group 1 received alternative phosphate sources, and Group 2 received a soluble phosphate source. The most significant morphological descriptor for Group 1 was SPAD (SPAD index). The mineral descriptor was potassium (K), and as for the results of the bromatological analysis, the crude protein (CP) content was the most significant. These descriptors showed lower values in treatments that received the soluble phosphate source. For Group 2, the most significant morphological descriptors were aerial fresh weight (AFW) and aerial dry weight (ADW), the mineral was calcium extract (ECa), and the bromatological descriptor was nitrogen extract (EN). Phosphorus was the 11th most significant descriptor in Principal Component 1 and the 12th most significant in Principal Component 2; thus, it had equal importance for both groups.

The *in vitro* tests showed that the PSI ranged from 2 to 3.61, and the phosphate-solubilizing bacteria belonged to *Bacillus*, *Enterobacter*, *Microbacterium*, *Micrococcus*, *Pantoea*, and *Pseudomonas*. These genera are already described in the literature as phosphate-solubilizing bacteria. De Assumpção et al. (2009) found *Pseudomonas* sp. with 5.3 and 8.3 of PSI and *Pantoea* sp. with PSI = 6.0. Suleman et al. (2018), when prospecting bacteria with the potential for phosphate solubilization for wheat plants, found that the best results were with *Enterobacter*, presenting 2.2–5.8 of PSI. Similarly, the strain with the highest PSI also belonged to the *Enterobacter* genus in the present study.

Regarding the *in vivo* test in a greenhouse, the treatments with crystalline phosphate plus Isolates 105 (*Pseudomonas* sp.) and 458 (*Pseudomonas* sp.) showed available phosphorus in the initial period of plant development. However, the treatment that received the 110 (*Enterobacter* sp.) strain showed higher phosphorus content in the second cut, suggesting that the 110 strain needs a more extended period to make the phosphorus available to the plant. In the literature, there is a search for an alternative source of phosphorus for plant nutrition, with Arad rock phosphate being one of those rocks with potential for this purpose. Gatiboni et al. (2003) used natural phosphates from Arad as a source of phosphorus for white clover (*Trifolium repens*) and ryegrass (*Lolium multiflorum*) pastures. Gatiboni et al. (2003) observed that the use of natural phosphate from Arad was effective in moderate to high soil and that liming increased the efficiency of superphosphate and decreased the efficiency of rock phosphate as a source of phosphorus. Guedes et al. (2012), evaluating the use of natural Arad phosphate and liming in two tropical grass species in degraded Amazon soil, observed better results in *Megathyrsus maximus* than in *Urochloa brizantha*, noting that the success in fertilization was dependent on the cultivated species and soil acidity. These studies sought to explore the gradual capacity of natural rocks to release phosphorus. However, they only used Arad’s natural phosphate in crops without studying the endophytic and rhizospheric microbiota of the host plant. In the present study, the primary method was the optimization of the phosphorus contained in the phosphate rock through selected microorganisms isolated from soil and plant tissue of *P. atratum*. Another characteristic observed in *P. atratum* was the acidic soil; both in the rainy and dry seasons, the soil pH of the soil was not higher than 5.6. As mentioned by Guedes et al. (2012), this feature facilitates the solubilization of phosphate from natural rocks.

Principal component analysis showed that the 110FCR (*Enterobacter* sp.) and 458FCR (*Pseudomonas* sp.) treatments were very close. The 105FCR (*Pseudomonas* sp.) treatment stood out, being the treatment with crystalline phosphate that came closest to the treatments that received soluble phosphate. The literature shows that the main phosphate solubilizers are *Arthrobacter*, *Azospirillum*, *Azotobacter*, *Bacillus*, *Beijerinckia*, *Burkholderia*, *Enterobacter*, *Erwinia*, *Flavobacterium*, *Mesorhizobium*, *Microbacterium*, *Pseudomonas*, *Rhizobium*, *Rhodococcus*, and *Serratia* (Bhattacharyya and Jha, 2012; Gouda et al., 2018). There is a search to optimize phosphate rock from Cajati as an alternative source of phosphorus. Lemos et al. (2013) sought to use the rock in the diet of Nellore cattle, as Bernardi and Oliveira (2021) sought to associate the phosphate rock of Cajati with zeolite minerals to nourish the alfalfa crop.

In treatments with sedimentary phosphate, the 109FSE (*Bacillus* sp.) treatment was the closest to the treatments with soluble phosphate and presented the highest phosphorus content in the first cut. One hypothesis to explain the higher content of phosphorus in the first cut is that there was great solubilization of sedimentary phosphate during the initial periods of the plant, thus depleting almost all available phosphorus sources in the first cut and, consequently, reducing these in dry matter in the second and third cut. This fact shows the importance of topdressing after grazing.

The results observed in treatments with soluble phosphate that included the bacterial isolates also suggest that the source of phosphorus was depleted during the initial stages of plant development. Of the eight treatments that received the strains, seven showed higher phosphorus content than the soluble phosphate control dry matter. The exception was treatment 103FSO (*Enterobacter* sp.). Therefore, future studies should explore the possibility of using less soluble phosphorus by inoculating phosphate-solubilizing bacteria, seeking to optimize the use of soluble phosphorus in agriculture. When looking for bacteria with the potential for phosphate solubilization in peas, Oteino et al. (2015) conducted an experiment involving *Bacillus* sp. and *Pseudomonas* sp. They used soluble phosphate as a control and tricalcium phosphate (Ca_3_(PO_4_)_2_) insoluble in the treatments. As a result, it was observed that the strains increased the phosphorus content in the plant compared to the insoluble control. Nevertheless, no treatment equaled or surpassed the phosphorus content present in the dry matter of the plants treated with soluble phosphate. Similar to the results presented by Oteino et al. (2015), no alternative treatments had equal or higher phosphorus levels as seen in treatments that received the soluble phosphate source. However, a significant difference was that treatments with soluble phosphate also received the bacteria, indicating the potential of these microorganisms to optimize the soluble phosphate in the plant.

During the *in vitro* tests, using the NFB medium, there was an expectation of finding *Azorhizophilus paspali* (*Azotobacter paspali*), a nitrogen-fixing bacteria, found by Döbereiner et al. (1995) when developing the culture medium (Baldani et al., 2014). Batista et al. (2018), when using NFB medium, observed that the main strains with potential for biological nitrogen fixation belonged to *Bacillus* and *Burkholderia*. As in these studies, no strains of *Azotobacter* were obtained in the present study. However, the growth of other microorganisms in the NFB medium was justified because the medium was not highly selective; therefore, strains with the potential to use malic acid as a carbon source and with a pH of 6.8 can grow (Baldani et al., 2014). This method characterized the strains with potential for nitrogen fixation in this work. The cultivable bacterial community with the potential for nitrogen fixation will undoubtedly increase by using another culture medium for isolation or biochemical characterization.

The bromatological descriptors crude protein (CP) and nitrogen (N) content correlated with the morphological descriptor index SPAD in the plant. The CP descriptor was calculated by multiplying the nitrogen content by 6.25 (Druzian et al., 2012; De Medeiros et al., 2015). The SPAD index was generated by the SPAD-502 chlorophyll meter (Soil Plant Analysis Development), which indirectly measures the leaf chlorophyll content without destroying the leaf (D’Oliveira et al., 2020), and the chlorophyll concentration positively correlated with the nitrogen content (Benati et al., 2021).

Among the most significant descriptors to assess the variation between treatments and plant growth promotion, crude protein content was significantly crucial in Group 1 treatments. The 161SF (*Pseudomonas* sp.), 103FSE (*Enterobacter* sp.), 25SF (*Pseudomonas* sp.), CAFCR, and 109SF (*Bacillus* sp.) were the ones with the highest SPAD index. On the other hand, the 103FCR (*Enterobacter* sp.), 25FCR (*Pseudomonas* sp.), 109SF (*Bacillus* sp.), 161SF (*Pseudomonas* sp.), and 105 FSE (*Pseudomonas* sp.) treatments presented the highest levels of CP and N.

Leite et al. (2001) studied the growth and chemical composition of *P. atratum* cv. Pojuca grass in soil with satisfactory nutrients and nitrogen fertilization during the rainy season. The researchers found CP contents between 6.90 and 12.11%. By sampling the nutritional contents of three cultivars, Porto et al. (2009) found CP contents of 11.1, 11.9, and 9.4% for Tanzania grass (*M. maximus*), Stargrass (*Cynodon*), and marandu grass (*U. brizantha* cv. Marandu), respectively.

Crude protein contents lower than 7% in the dry matter limit animal nutrition (De Abreu et al., 2006). All treatments studied in the present work had a protein content greater than 7%, ranging from 10.12 to 12.46%, values similar to those found by Leite et al. (2001) for the Pojuca cultivar (*P. atratum*) and by Porto et al. (2009) for the cultivars Capim-tanzânia, Grama-estrela, and Capim-marandu. The leaf protein content in the Pojuca cultivar ranges from 8 to 10% (Karia and de Andrade, 2001). The values found in this work were also superior to the results obtained by Lopes et al. (2010) for *U. brizantha*, *U. decumbens*, *U. humidicola,* and *U. Ruziziensis,* which ranged from 6.4 to 7.5% CP in dry matter.

In the present study, none of the treatments received nitrogen fertilization. Nevertheless, many of them had similar or superior CP results compared to other studies that evaluated cultivars already on the market. Even the phosphate-free control (CSF), which did not receive any phosphorus source or bacterial inoculum, showed CP results superior to those found by Leite et al. (2001). This fact shows the potential of this genotype as a forage plant.

The main potential NFB were 25 (*Pseudomonas* sp.), 103 (*Enterobacter* sp.), 105 (*Pseudomonas* sp.), 109 (*Bacillus* sp. and 161 (*Pseudomonas* sp.). The 103FSO (*Enterobacter* sp.) treatment stood out due to high levels of CP and N in the dry matter. The Isolate 103 (*Enterobacter* sp.) also showed good levels of CP and N in treatments with a sedimentary and crystalline phosphate source, making it a good candidate for biological nitrogen fixation investigation. Potassium was another significantly important mineral descriptor observed in Group 1 treatments. In the K^+^ format, potassium regulates the osmotic potential and activates enzymes involved in respiration and photosynthesis in the plant (Taiz et al., 2016). Therefore, potassium was among the most significant descriptors in the principal component analysis. The treatments that presented the lowest K content received the soluble phosphate source. The treatments that showed the highest K content were 105FSE (*Pseudomonas* sp.), 103FSE (*Enterobacter* sp.), 170FSE (*Pantoea* sp.), 25FCR (*Pseudomonas* sp.), and 161FCR (*Pseudomonas* sp.), followed by the control treatments CASF, CAFCR, and CAFSE. The microbiological modification technique enables the direct application of rocks in agriculture. Citric and oxalic acids produced by microorganisms release potassium from biotite, a common mineral in the silicate class (Van Straaten, 2010).

The plants inoculated with the bacterial Isolates 105FCR (*Pseudomonas* sp.), 109FSE (*Bacillus* sp.), 110FSE (*Enterobacter* sp.), 103SF (*Enterobacter* sp.), 458SF (*Pseudomonas* sp.), and 458FCR (*Pseudomonas* sp.) showed growth similar to those treated with a soluble phosphate source. Therefore, they can be selected for future plant growth-promotion experiments.

## Conclusion

A total of 116 cultivable endophytic and rhizospheric strains were isolated from rhizospheric soil samples, roots, and leaves of *P. atratum*.

As for the capacity of the plant growth-promoting bacterial strains, 43 (37.00%) strains showed positive NFB, SF, and IAA results and belonged to *Enterobacter* (46.50%), *Pseudomonas* (32.50%), and *Pantoea* (13.90%), and *Bacillus*, *Microbacterium*, and *Micrococcus* strains represented 6.90%.

The phosphate solubilization index (PSI) ranged from 2 (*Pseudomonas* spp.) to 3.61 (*Enterobacter* spp.) and the IAA production ranged from 12.85 (*Pseudomonas* spp.) to 431.41 (*Pantoea* spp.) μg ml^−1^.

In the *in vivo* test, treatments 105 (*Pseudomonas sp*.) and 458 (*Pseudomonas sp*.) were the most significant for the crystalline phosphate source, 109 (*Bacillus sp*.) for the sedimentary phosphate source and, as for the soluble phosphate source most treatments that received bacterial isolates had higher phosphorus content in the dry matter than the uninoculated soluble phosphate control.

For the morphological, mineral and bromatological variables, the principal component analysis showed that Principal Component 1 had significant associations with the SPAD index (SPAD), potassium (K), zinc (Zn), crude protein (CP), lignin (LIG) and nitrogen (N). While, principal Component 2 showed strong associations with the other 16 descriptors.

These diverse cultivable bacterial genera have the potential to promote plant growth, and the 105FCR (crystalline phosphate + *Pseudomonas* sp.), 109FSE (sedimentary phosphate + *Bacillus* sp.), and 110 FSE (sedimentary phosphate + *Enterobacter* sp.) treatments showed the best results in the plant growth promotion assay.

Other treatments showed isolated characteristics of interest for one or another descriptor analyzed, such as dry weight, potassium, and nitrogen content in the leaves.

## Data availability statement

The datasets presented in this study can be found in online repositories. The names of the repository/repositories and accession number(s) can be found at: https://www.ncbi.nlm.nih.gov/genbank/, MK521286, MK521276, MK521285, MK521301, MK521314, MK521306, MK521273, MK521304, MK521284, MK521289, MK521303, MK521302, MK521274, MK521300, MK521275, MK521296, MK521290, MK521293, MK521287, MK521271, MK521270, MK521283, MK521282, MK521272, MK521279, MK521308, MK521277, MK521297, MK521278, MK521292, MK521299, MK521291, MK521280, MK521298, MK521281, MK521305, MK521307, MK521261, MK521288, MK521263, MK521262, MK521264, and MK521265.

## Author contributions

All authors listed have made a substantial, direct, and intellectual contribution to the work and approved it for publication.

## Funding

This work was supported by grants from the São Paulo Research Foundation, FAPESP (Proc. No. 2020/11315-6).

## Conflict of interest

ND, WJ, AB, BV, and AF were employed by Embrapa Pecuária Sudeste.

The remaining authors declare that the research was conducted in the absence of any commercial or financial relationships that could be construed as a potential conflict of interest.

## Publisher’s note

All claims expressed in this article are solely those of the authors and do not necessarily represent those of their affiliated organizations, or those of the publisher, the editors and the reviewers. Any product that may be evaluated in this article, or claim that may be made by its manufacturer, is not guaranteed or endorsed by the publisher.

## Footnotes

^1^ https://www.embrapa.br/en/pecuaria-sudeste
